# Similarity in Recombination Rate Estimates Highly Correlates with Genetic Differentiation in Humans

**DOI:** 10.1371/journal.pone.0017913

**Published:** 2011-03-28

**Authors:** Hafid Laayouni, Ludovica Montanucci, Martin Sikora, Marta Melé, Giovanni Marco Dall'Olio, Belén Lorente-Galdos, Kate M. McGee, Jan Graffelman, Philip Awadalla, Elena Bosch, David Comas, Arcadi Navarro, Francesc Calafell, Ferran Casals, Jaume Bertranpetit

**Affiliations:** 1 IBE, Institute of Evolutionary Biology (UPF-CSIC), CEXS-UPF-PRBB, Barcelona, Catalonia, Spain; 2 National Cancer Institute, National Institutes of Health, Frederick, Maryland, United States of America; 3 Department of Statistics and Operations Research, Universitat Politècnica de Catalunya, Barcelona, Spain; 4 Faculty of Medicine, Ste-Justine Hospital Research Centre, University of Montreal, Montreal, Quebec, Canada; 5 Institució Catalana de Recerca i Estudis Avançats, Barcelona, Catalonia, Spain; 6 National Institute for Bioinformatics (INB), Barcelona, Spain; Institut de Biologia Evolutiva - Universitat Pompeu Fabra, Spain

## Abstract

Recombination varies greatly among species, as illustrated by the poor conservation of the recombination landscape between humans and chimpanzees. Thus, shorter evolutionary time frames are needed to understand the evolution of recombination. Here, we analyze its recent evolution in humans. We calculated the recombination rates between adjacent pairs of 636,933 common single-nucleotide polymorphism loci in 28 worldwide human populations and analyzed them in relation to genetic distances between populations. We found a strong and highly significant correlation between similarity in the recombination rates corrected for effective population size and genetic differentiation between populations. This correlation is observed at the genome-wide level, but also for each chromosome and when genetic distances and recombination similarities are calculated independently from different parts of the genome. Moreover, and more relevant, this relationship is robustly maintained when considering presence/absence of recombination hotspots. Simulations show that this correlation cannot be explained by biases in the inference of recombination rates caused by haplotype sharing among similar populations. This result indicates a rapid pace of evolution of recombination, within the time span of differentiation of modern humans.

## Introduction

The recombination rate is neither constant along chromosomes nor across species. The rate within genomes has been observed to vary at both the megabase level, with different chromosomal regions in the human genome showing differences in their recombination rates [1], [2] and at a finer level, due to the existence of recombination hotspots [2], [3], [4]. Comparisons of the human and the chimpanzee genomes have revealed poor conservation of recombination landscapes, in contrast to the high level of DNA sequence conservation observed among these species [5], [6]. Multiple lines of evidence suggest that sequence changes in the zinc-finger protein PRDM9 may be responsible for hotspots differences among species [7], [8]. Recombination rates have also been compared among human populations, revealing large-scale conservation [9], while some differences in hotspot intensities and some population-specific hotspots have been described at a finer scale [3], [10], [11], [12], [13]. Finally, different studies have shown the existence of individual variation in recombination [14], [15] and its heritability has been investigated, along with its biological consequences [16].

Measuring the fine-scale recombination rate is experimentally challenging, the resolution of recombination maps experimentally obtained is limited by relatively few meioses and a low density of markers, exception made by the recent paper by Kong et al. [17]. However good estimates can be obtained by applying population-genetic methods to DNA sequences [18]. Statistical methods have been developed to infer the fine-scale structure of recombination rate variation from genome-wide scale data [4]. One of the widely used methods is implemented in the LDhat package, which is based on a composite-likelihood approach. Simulations have shown that LDhat produces largely unbiased rate estimates of the fine-scale genetic map [19]. More recently, Khil and Camerini-Otero [20] have shown that present-day genetic crossovers are well predicted by a population averaged hotspot map computed from linkage disequilibrium data.

Differences in recombination rates among human populations provide a useful temporal framework to analyze the evolution of the recombination landscape, as they are recent enough to capture fast evolutionary changes. The basal branches of the genetic diversification of human populations happened some 150,000 years ago, a much shorter time than the split between humans and chimpanzees (around 6.5 million years). The comparison of the recombination patterns among human populations provides a means to verify whether recombination landscapes evolve over time. To address this issue, we analyzed whether differences in recombination rates among human populations are correlated with their genetic differences computed as genetic distances. Whole genome estimations of recombination rates based on SNP data are already available for HapMap samples which, however, consist only of four populations for HapMap Phase I and II [21], [22] and 11 populations for HapMap Phase III [23]. Here we computed the recombination rates using data for 660,918 SNPs on the Illumina HumanHap650K Beadchips genotyped in the full HGDP-CEPH panel samples [24], [25] for 28 populations belonging to six continental groups representing most of the worldwide human diversity [26].

## Materials and Methods

### Recombination rate estimation

We considered the H971 subset of the Human Genome Diversity Cell Line Panel (HGDP-CEPH) recommended by Rosenberg [27]. The 51 original HGDP-CEPH population samples [26] were re-grouped into 39 populations based on geographic and ethnic criteria as in [28]. To avoid small sample sizes, the analysis was performed on genotypes from 28 populations belonging to six continental groups, with sample sizes over 19 individuals (a list of used populations and their number of individuals is presented in Table 1). We used data for 660,918 SNPs on the Illumina HumanHap650K Beadchips successfully genotyped in the full HGDP-CEPH panel samples [24], [25]. SNPs are spaced 4.4 kb apart on average, an appropriate length given that hotspots occur every 200 Kb or less and their widths are 1–2 Kb [4], [29]. Population recombination rates were calculated between neighboring SNPs according to the method implemented in the *rhomap* program [30] within the LDhat package [31]. LDhat methods have been demonstrated to give highly similar results to alternative approaches in human and chimpanzee datasets [6], [29] and are computationally practicable for genome wide variation surveys. For a reliable estimation of the recombination rates, loci with more than 10% missing data in at least one population were discarded from the analysis. After this cleaning procedure, the total number of SNPs included in the analysis was 636,933 (96% of all the SNPs in the HGDP). The number of SNPs for each chromosome is reported in Table 2. For each population, 5 independent runs of the *rhomap* program were carried out (with parameters: iterations = 10.000.000, sampling = 5.000, burnin = 100.000). For each pair of adjacent SNPs we obtained 5 estimates of the population recombination rate (*4N_e_r*/kb) in each population and the median of these 5 estimates was used in the analysis.

**Table 1:** Mean recombination rates (4Ner/kb) corrected for effective population size with the standard deviation for all populations and their number of individuals.

	Population	Mean	SD	Number of individuals
**SSAFR**	Yoruba	0.0209	0.0176	21
	Biaka pygmies	0.0188	0.0164	21
	Mandenka	0.0213	0.0179	22
	Bantu	0.0211	0.0173	19
**Europe**	French	0.0214	0.0221	28
	Basque	0.0205	0.0210	24
	Russian	0.0211	0.0212	25
	North Italy	0.0218	0.0207	20
	Sardinian	0.0204	0.0216	28
**MENA**	Druze	0.0192	0.0221	42
	Bedouin	0.0198	0.0218	46
	Palestinian	0.0203	0.0224	46
	Mozabite	0.0222	0.0208	29
**Central South Asia**	Brahui	0.0220	0.0213	25
	Balochi	0.0222	0.0213	24
	Hazara	0.0217	0.0206	22
	Burusho	0.0220	0.0216	25
	Kalash	0.0183	0.0193	23
	Makrani	0.0222	0.0211	25
	Pathan	0.0220	0.0209	22
	Sindhi	0.0228	0.0215	24
	North West China	0.0203	0.0215	29
**East Asia**	Han	0.0139	0.0203	44
	Japanese	0.0166	0.0200	28
	North East China	0.0164	0.0210	36
	South China	0.0102	0.0177	66
	Yakut	0.0181	0.0200	25
**America**	Maya	0.0162	0.0188	21

**Table 2:** Mantel's *r* correlation per chromosome.

Chromosome	Mantel's *r*	Number of SNPs
1	0.909	49,162
2	0.909	53,187
3	0.853	44,049
4	0.897	39,439
5	0.911	40,579
6	0.932	42,699
7	0.893	35,076
8	0.850	36,850
9	0.893	30,815
10	0.946	34,124
11	0.922	31,660
12	0.891	31,494
13	0.878	24,918
14	0.851	21,241
15	0.884	19,381
16	0.761	19,515
17	0.887	16,427
18	0.876	19,948
19	0.805	10,576
20	0.875	16,764
21	0.879	9,523
22	0.820	9,506

All values were significant at *P<*0.0001. Number of iterations: 9,999.

Since population recombination rates (ρ) are dependent on the effective population size (ρ* = 4N_e_r*), estimates of the population recombination rate in each population were normalized by *θ = 4N_e_µ*, a scaled population mutation rate obtained from the same individuals and populations, where *µ* is the genome-wide average microsatellite mutation rate per locus and per generation [13]. We have used a measure obtained though microsatellites because they represent a totally independent set of data and thus there will not be problems of circularity; moreover they refer to exactly the same populations. As there is no evidence of mutation rates varying among human groups, this correction produces values that are not biased by effective population size.

### Correlation between genetic distance and recombination dissimilarity

We obtained a Spearman rank correlation matrix for the recombination rates among all pairs of populations. Each correlation value was obtained by comparing the values of corrected ρ (see above) for all pairs of adjacent typed SNPs between a population pair. In order to simplify the comparison with the genetic distance, the Spearman correlation values were turned into a dissimilarity measures by subtracting them from 1. The obtained 28×28 matrix is then a measure of the dissimilarity of recombination rates between each pair of populations.

The differentiation among human populations was estimated through the F_ST_ measure [32] among each pair of populations. F_ST_ values were calculated using a routine implemented in the PopGen module of BioPerl [33] and stored in a 28×28 matrix.

The matrix of recombination dissimilarity and that of genetic distance (F_ST_ matrix), were compared using a standardized Mantel test [34] by randomly permuting 9,999 times the rows and columns of one of the matrices. Statistical analyses were implemented using the R statistical software.

### Simulation analysis

To further investigate the effect of the sharing of haplotypes and, hence of linkage disequilibrium patterns (which are at the base of the recombination rate estimates) on the relationship between genetic distance and recombination landscape, we designed a simulation study. We simulated human demography under a model in which the recombination rate was the same for all the simulated populations, and we sought to determine whether the correlation between genetic distance and inferred recombination similarity between simulated populations was similar to the observed in empirical data.

The simulations were carried out with the COSI program [35] which provides a simulation of the human demography under a three-population model based on the HapMap populations. This model was specifically designed to generate sequences that closely resemble empirical data of three human populations (African, European and Asian) by means of simulating a human-like demography and a variable recombination rate along the sequences, allowing for presence and absence of hotspots. Cosi consists of two programs which are run one after the other. The first generates a random local recombination map based on the distribution seen in the deCODE genetic map for the autosomes [1]. The second, is the coalescent program itself and it builds up a coalescent network taking into account the local recombination map generated previously. Therefore, each simulation will generate a different recombination landscape with different number of hotspots and coldspots. Specifically, the model was calibrated to obtain realistic F_ST_ values that mimic the divergence found among the three human populations being simulated and to obtain similar values of the frequency distribution of alleles, among other parameters. We performed 1000 simulations using the best-fitting demographic model provided by COSI. For each simulation, we set the length of the simulated sequences to 1 Mb and adopted a sample size of 56 sequences for European and Asian populations and 42 for the African population with the aim of having the same amount of individuals as in a three chosen equivalent HGDP populations (Yoruba, French and Japanese). In each simulation, the distribution of the recombination rate is the same for the three simulated populations; this leads to simulated genotypes of different populations that share common haplotypes but have not experienced differences in their recombination rate. Finally, as SNPs included on Illumina's HumanHap650Y Genotyping BeadChip are tagSNPs with r^2^ higher than 0.8, and in order to have a similar ascertainment bias in the simulations and in the observed data, we performed a selection of tagSNPs with the same criteria using Haploview software with the pairwise option [36].

In order to compare simulated data to a consistent empirical dataset, we randomly chose, along the whole genome, 1000 non-overlapping 1 Mb long windows, and we analyzed them across the three populations of Yoruba, French and Japanese.

We then computed F_ST_ and recombination rates, following the same procedure as before, for real and simulated data. If the shared haplotypes were the main source of the high correlation found between recombination and genetic distance, we expect to observe this correlation also in the simulated data.

## Results and Discussion

### Exploratory analysis of recombination rates

Population recombination rates were computed between 636,933 neighboring SNPs for 28 populations. As the recombination rate was estimated through several runs for each population, and to test for the agreement of estimates between runs of the same chromosome, 10 runs were performed for chromosome 22 for all populations. We carried out a repeated measure ANOVA testing population and run as the main effects and pairs of adjacent SNPs as a covariate. No statistical significance of runs was found, but population and pairs of adjacent SNPs were highly significant (data not shown). This result reflects that the noise in the estimation procedure is low in relation to differences between populations.

**Figure 1:**
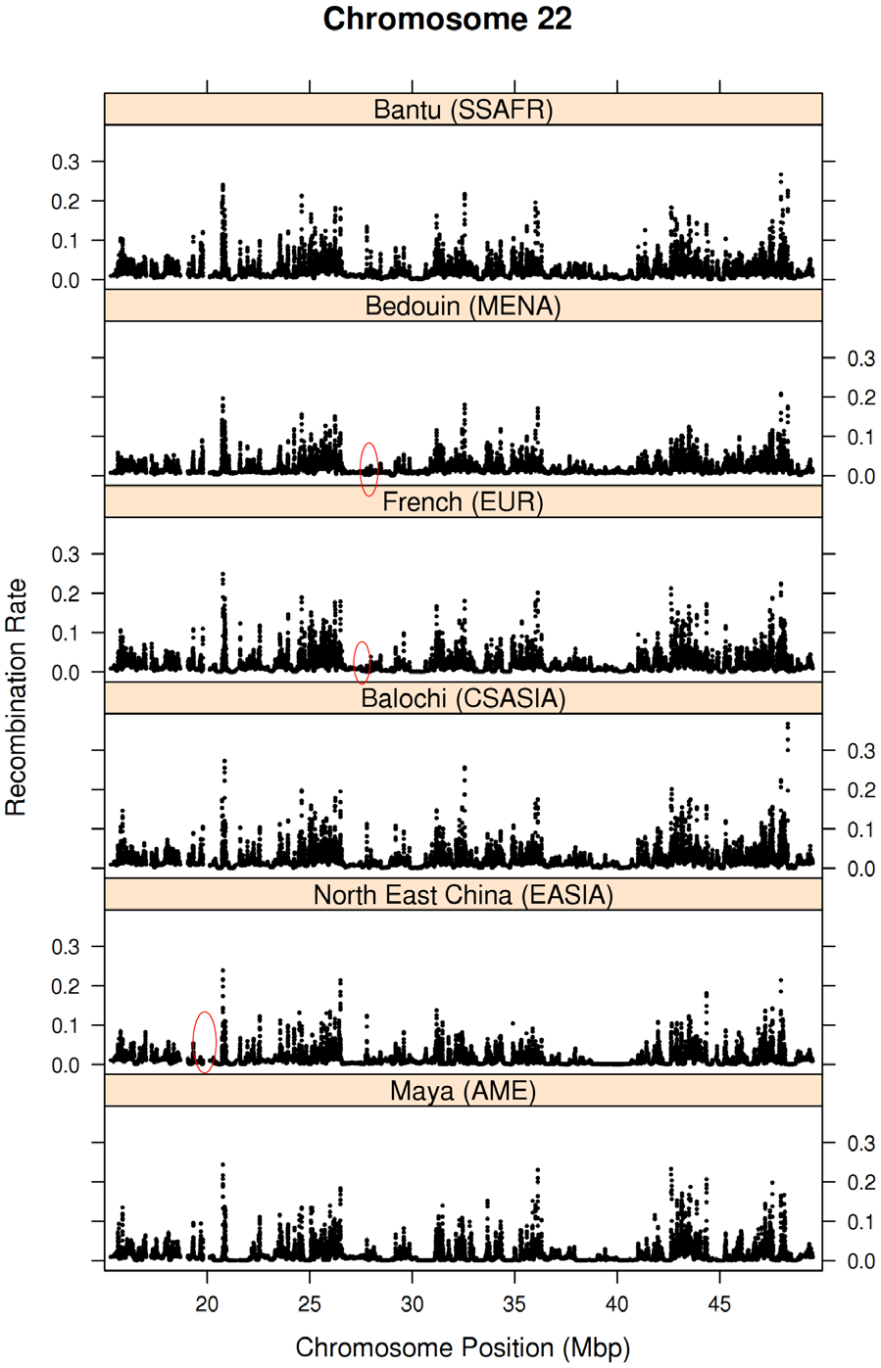
Recombination rate estimates (4Ner/Kb) corrected for effective population size for successive SNP-pairs for chromosome 22 for 6 populations.

Table 1 shows the mean estimated recombination rate for all populations, grouped according to their geographical region. Results indicate considerable variation in recombination rates between populations, with low recombination rates for populations from East Asia. A repeated measure ANOVA shows that differences between populations are highly significant (F_27, 636,910_ *= *59,479.8; *p<*0.00001; R^2^ *= *8.5%). A Friedman ANOVA test shows similar results (ANOVA χ^2^ *= *2,255,369; *p<*0.000001; df = 27). Post hoc analysis using a Bonferroni correction for the repeated ANOVA test show that differences between populations remain significant, except for two homogenous groups from Central South Asia: Pathan, Burusho and Brahui on the one hand; and Mozabite, Balochi and Makrani on the other. Figure 1 shows the estimated recombination rate (scaled by the genome-wide average microsatellite mutation rate) along chromosome 22 for 6 populations (one from each continental region; all populations are shown in Figure S1). The figure shows a similar pattern for all populations; but substantial variation can be detected by close observation. For example, North East China and Maya exhibit fewer hotspots than the other populations. A hotspot located just before 20 Mbp in all populations is absent (or much weaker) in North East China. A hotspot region located around 25 Mbp is absent (or much weaker) in Bedouin and French, but present in other populations. More variation is observed when considering all populations (Figure S1). This variation is consistent with previous reports in other genomic loci and genome-wide [13].

### Genetic distance and recombination similarity between populations

**Figure 2:**
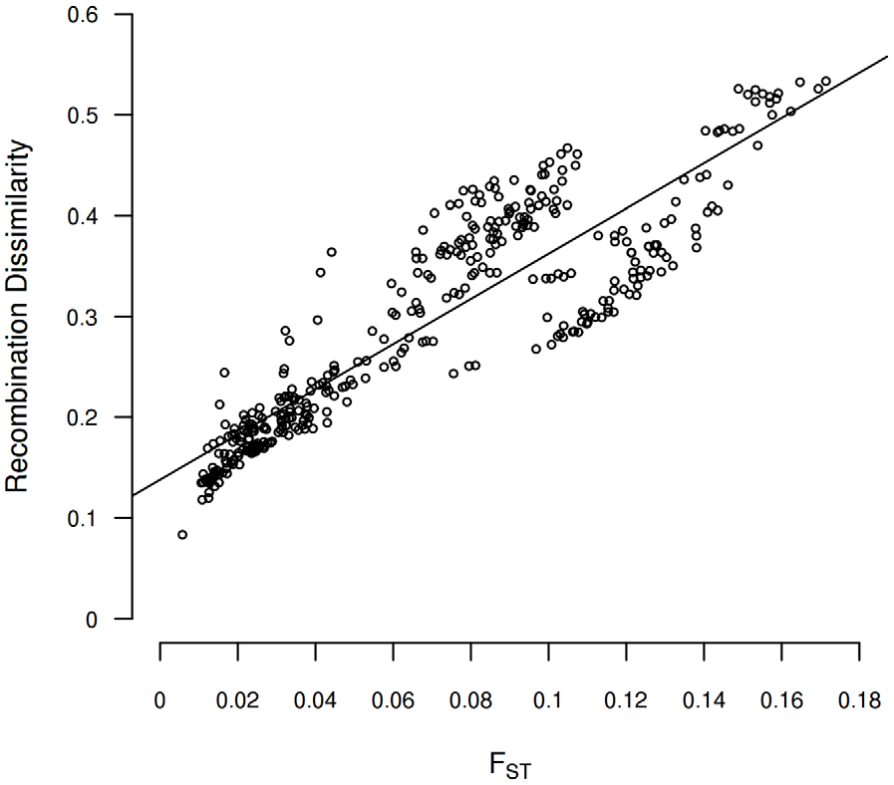
Relationship between F_ST_ values and the recombination rate correlation based on 378 pairwise populations comparisons.

Spearman rank correlation between population recombination estimates were obtained by comparing the values of corrected recombination ρ/θ for all pairs of adjacent typed SNPs between each population pair. The differentiation among human populations was estimated through the F_ST_ measure [32] among each pair of populations. The correlation values in recombination between population pair and F_ST_ measures were stored as dissimilarity and distance matrices respectively and compared using a standardized Mantel test [34]. A significant Mantel's *r* correlation of 0.894 (*p<*0.0001) was observed, indicating that differences in recombination rates among populations increase with their genetic distance (Figure 2). In other words, genetic differentiation across human populations explains a considerable amount of recombination differences among them. This result also stands when the analysis is independently performed for each chromosome; then the Mantel test correlation ranges from 0.761 for chromosome 16 to 0.946 for chromosome 10 (Table 2).

**Figure 3:**
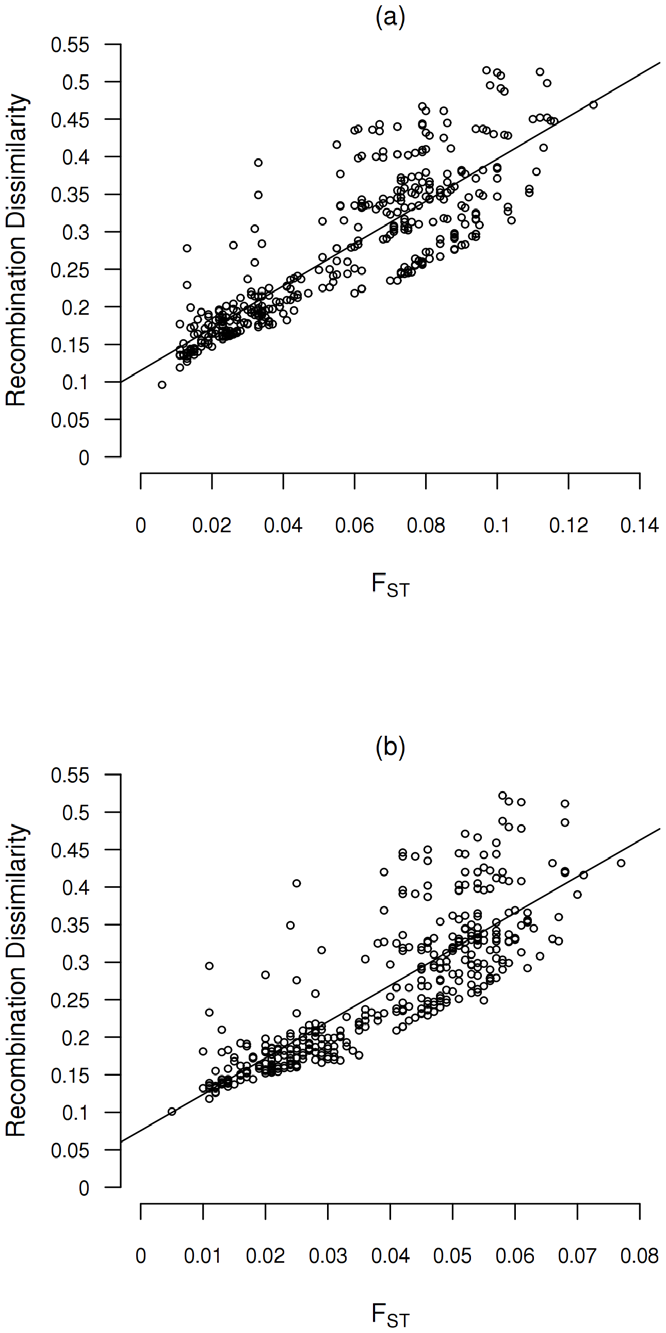
Relationship between F_ST_ values and the recombination rate correlation for SNPs with a) MAFs higher than 0.1 and b) MAFs higher than 0.2.

Genetic differentiation estimated through F_ST_ is a measurement of allele frequency differences. LD based estimation of recombination rate is also affected by allele frequencies at the marker loci. To explore the effect of allele frequency on recombination estimates, SNPs were placed into 10 bins based on their minor allele frequency calculated using the global allele frequency of all populations together. An analysis of variance using recombination estimates as dependent variable and MAFs as a mean effect shows significant differences between categories for all populations, with bins with high MAFs showing high recombination estimates (F_9, 636,901_ *= *776,8 p = <0.0001; Figure S2). However no differences were observed between bins with MAFs higher than 0.2. The correlation between genetic differentiation and recombination dissimilarity using only SNPs within each MAFs category remains very high and strongly significant (Table 3). The mean Mantel's *r* correlation from the different MAFs categories is slightly smaller than the one obtained from individual chromosomes using all SNPs together (0.858 and 0.878 respectively). We also calculated this correlation using only SNPs with a minor allele frequency (MAFs) above 0.1 and above 0.2 in the 28 analyzed populations. This analysis includes 141,921 SNPs and 34,706 SNPs respectively and show similar results to the mean analysis (Figure 3), the Mantel's *r* correlation is 0.855 for SNP with MAFs high than 0.1 and 0.830 for SNPs with MAFs high than 0.2 and highly significant for both (*p<*0.00001).

**Table 3:** Mantel's *r* correlation per minor allele frequency (MAF) bins.

MAF	Mantel's *r*	Number of SNPs
≤0.05	0.741	72,117
0.05<MAF≤0.10	0.866	67,883
0.10<MAF≤0.15	0.917	72,455
0.15<MAF≤0.20	0.923	70,741
0.20<MAF≤0.25	0.910	66,872
0.25<MAF≤0.30	0.886	62,211
0.30<MAF≤0.35	0.862	59,298
0.35<MAF≤0.40	0.846	56,427
0.40<MAF≤0.45	0.824	54,973
0.45<MAF≤0.50	0.805	53,943

All values were significant at *P<*0.0001. Number of iterations: 9,999.

It can be argued that these results could be driven by a bias in the selected populations, that is, by similar recombination rates in populations belonging to the same continental group, due to the presence of common or shared haplotypes, and - not to smaller changes in crossing-over rate. To test this hypothesis, we repeated our analysis considering only one population per continental group, thus avoiding redundancy in the genetic composition of the populations in our dataset. In particular, the analysis was performed with data from Yoruba (Africa), French (Europe), Bedouin (Middle East/North Africa), Burusho (Central/South Asia), Han (East Asia) and Maya (America) populations. The observed correlation remained very high (Mantel's *r = *0.863, *p = *0.002) and was statistically significant even with the low number of pairwise comparisons.

To test for the impact of using the same data set for estimating recombination and genetic distance, we performed a Mantel test between the F_ST_ matrix calculated for each individual chromosome versus the recombination dissimilarity matrix computed on all the other chromosomes. This makes the estimates of recombination and genetic distance effectively independent since they are estimated from different parts of the genome, and avoids any problem of circularity in our analysis. Results are presented in Table S1. The correlation remains and is still highly significant in all cases (*p<*0.00001).

### Hotspots analysis

Alternatively, comparisons of recombination rates among populations can be evaluated by attending to the presence or absence of recombination hotspots. This analysis has a special value as it is much less dependent on the accuracy of estimating the recombination rates. We defined a hotspot in each population as a recombination rate that exceeds 5 times the mean rate, with a threshold of 0.1×4Ner/Kb. A total of 22,413 hotspots have been detected at least in one population each. The number of hotspots vary among populations, from 2,582 for South China to 8,042 for Palestinian (no correlation between the number of hotspots and population sample size was observed, Pearson correlation test *r = *−0.08 *p*>0.05; Spearman correlation test *r = *0.34 *p*>0.05).

Taking into account only the common hotspots shared by all populations within a given continental region, the proportion of shared hotspots between continental regions is maximum between Europe and Middle East and North Africa (0.52), Europe and Central South Asia (0.44) and between Middle East and North Africa and Central South Asia (0.41). These values are, as expected, much lower when considering Sub-Saharan African or East Asian populations (Table 4). An interesting outcome from this analysis is the number of hotspots common to non African human populations compared to Sub-Saharan Africans. The proportion of hotspots shared between these two groups is only 17.4%, which is a small proportion given the recent out of Africa origin of non African population, and also show that the pace of evolution of hotspots is substantial. Figure S3 shows, as an example, patterns of recombination rates for SNPs where a hotspot event was detected in at least one population. Most variation is observed between continental groups while there is a substantial pattern sharing among populations belonging to the same continental group.

**Table 4:** Number of fixed hotspots (diagonal, bold) within a continental region, common hotspots shared between a pair of continental regions (upper, italics) and the proportion of shared hotspots in relation to the fixed hotspots (lower).

	SSAFR	MENA	EUR	CSASIA	EASIA
**SSAFR**	**1870**	*1212*	*1146*	*967*	*527*
**MENA**	0.29	**3473**	*2241*	*1597*	*990*
**EUR**	0.30	0.52	**3048**	*1539*	*900*
**CSASIA**	0.33	0.41	0.44	**1984**	*806*
**EASIA**	0.18	0.24	0.24	0.29	**1575**

SSAFR, MENA, EUR, CSASIA and EASIA stand respectively for Sub-Saharan Africa, Middle East and North Africa, Europe, Central South Asia and East Asia.

We calculated the Jaccard distance between each pair of populations to measure the overall difference in presence/absence of hotspots (defined as the size of the intersection divided by the size of the union of sample sets; in this distance the absence of a hotspot in a given position in two populations does not contribute to the similarity between them as would be in the case of a simple matching coefficient). Comparing this distance matrix with the F_ST_ matrix, highly significant results were obtained (Mantel's *r = *0.866, *p<*0.0001), suggesting that differences in the location of recombination hotspots increases with genetic differentiation between human populations.

### Simulation analysis

With the Mantel test analysis using only one population from each continent, we have shown that the effect of haplotype sharing in closely related populations does not explain the correlation between genetic differentiation and recombination. However, it is possible that the sharing of haplotypes and, hence of linkage disequilibrium patterns, had a considerable effect also on distant populations, since its origin can be traced back to the Out of Africa origin of modern humans. To disentangle this point, we performed a simulation study designed to recognize the impact of using shared haplotypes on the estimates of recombination rates.

As the number of simulated populations is only three, the Mantel test cannot provide a robust comparison. To compare the relationship between recombination similarities and genetic differentiation in the three populations being simulated and in the three corresponding HGDP populations, we performed a Spearman correlation of the values of recombination between all neighboring SNPs in the 1Mbp region and their F_ST_ values, for both simulated and empirical data. This is a more stringent test than the previous overall comparison between F_ST_ and recombination patterns, since, rather than general means, data points correspond now to 1000 windows a 1 Mb each. The correlation between recombination values and genetic distance for empirical data are 0.26, 0.25 and 0.27 for Yoruba-French, Yoruba-Japanese, and French-Japanese respectively (all significant). The values here are much lower than before as they refer to correlations between F_ST_ and the correlation of the recombination values for windows of 1 Mb in two populations and not to distances (in the Mantel test, two matrices of F_ST_ and recombination dissimilarity between points of populations were compared). Conversely, these values are only 0.05, 0.06 and 0.09 for the simulated African-European, African-Asian and European-Asian (only the last comparison was marginally significant). This shows that, within the simulated populations, F_ST_ and recombination rate were not correlated despite sharing common haplotypes, whereas they are clearly correlated within the three studied populations. The common origin of haplotype structure, as illustrated in the simulation data, is unlikely to have contributed to a large part of the correlation between genetic distances and structure of the recombination landscape.

In the simulations we have not considered the possible impact of natural selection and its consequences both on the estimated taxes of recombination (as they rely on measures of linkage disequilibrium) and on F_ST_ measures. As its impact is likely to be restricted to the relative low number of regions that could be under very recent positive selection and acting differentially among populations [37], it is unlikely to have a genome wide impact. Nonetheless, the relationship between recombination, population differentiation and selection in humans is still a working and open field.

### Concluding remarks

The results of this study reveal the footprint of the evolutionary history of human populations on the recombination rate. The large differences found in the comparison of the recombination landscapes among humans and chimpanzees [5], [6] showed that recombination evolves quickly. Here, we give evidence that, even at the narrow timescale separating human populations, on the order of tens of thousands of years, differences appear to be detectable and to be correlated with genetic differentiation among populations. Recombination rate appears to be a rapidly changing parameter, indicating that the underlying factors shaping the likelihood of a recombination event, such as DNA sequences controlling recombination rate variation, also change. The change is strongly detectable also in terms of presence or absence of recombination hotspots even if at the present stage it is not possible to measure the relative importance between changes in intermediate recombination rates and the appearing or disappearing of recombination hotspots. This is consistent with recent data showing that allelic variants of PRDM zinc fingers are significantly associated with variability in genome hotspots among humans [8]. The results obtained in this work contribute to the growing perception of recombination not as fixed feature of the genome of a species, but as a phenotype with ample genetic variation.

## Supporting Information

Figure S1 **Recombination rate estimates (4Ner/kb) corrected for effective population size for successive SNP-pairs for chromosome 22 and in each of 28 populations, grouped into geographical regions.**
(PDF)

Figure S2 **Mean of the recombination estimate (4Ner/kb) for all populations calculated for 10 categories of SNPs based on their minor allele frequency.** MAFs are calculated using the global allele frequency of all populations together.
(TIF)

Figure S3 **Heatmap showing patterns of hotspots observed for 300 SNPs of chromosome 22 for the 28 populations, grouped according to their geographical region.** The first 300 SNPs of chromosome 22, for which a hotspot is present in at least one population, are reported on the x axis. In color, for each population the value of the recombination estimate (4Ner/kb) corrected for effective population size for that SNP in a gradient from blue (low recombination values) to green (high recombination values).
(TIF)

Table S1 **Mantel's** ***r*** **values between the genetic distance and recombination dissimilarity matrices.** First row shows chromosome for which the genetic distance was calculated; first column show the chromosome for which the recombination matrix was calculated. All value were highly significant (*p*<0.00001).
(DOC)
