# Supplementary figures and images for: Similarity in Recombination Rate Estimates Highly Correlates with Genetic Differentiation in Humans
Source: PLoS One. 2011 Mar 28;6(3):e17913. doi: 10.1371/journal.pone.0017913 (PMC3065460; doi:10.1371/journal.pone.0017913)

# Chromosome 22

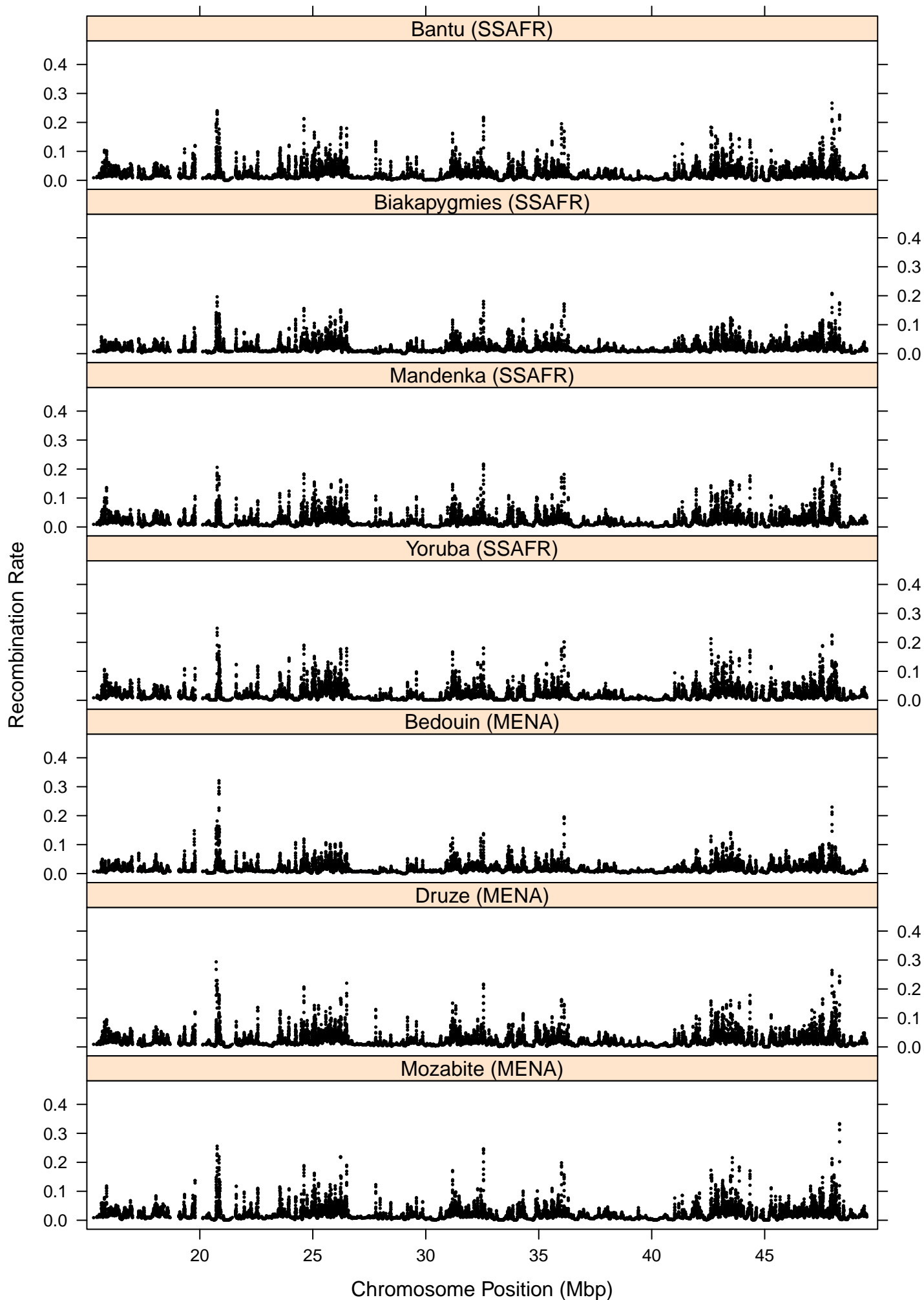

# Chromosome 22

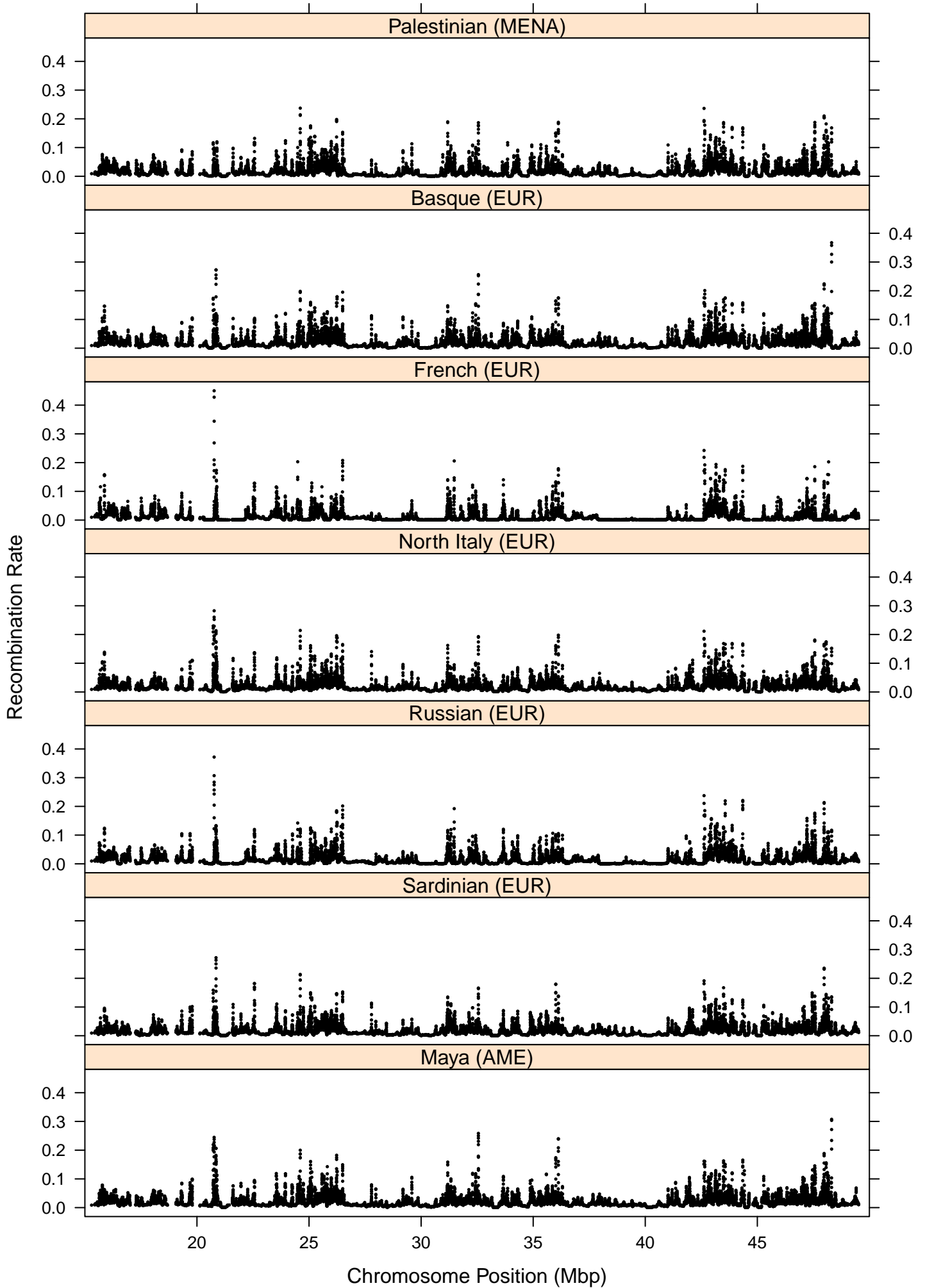

# Chromosome 22

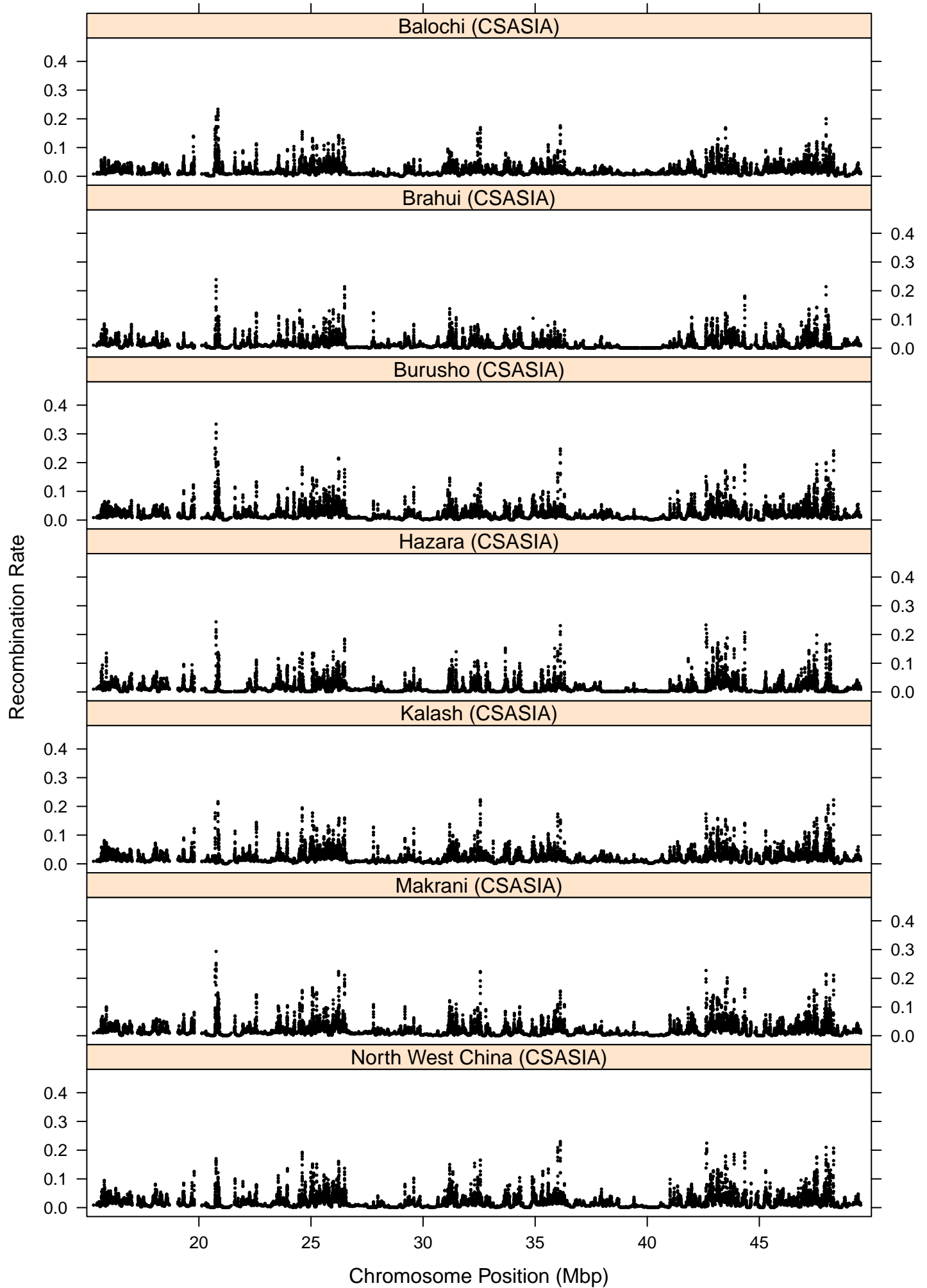

# Chromosome 22

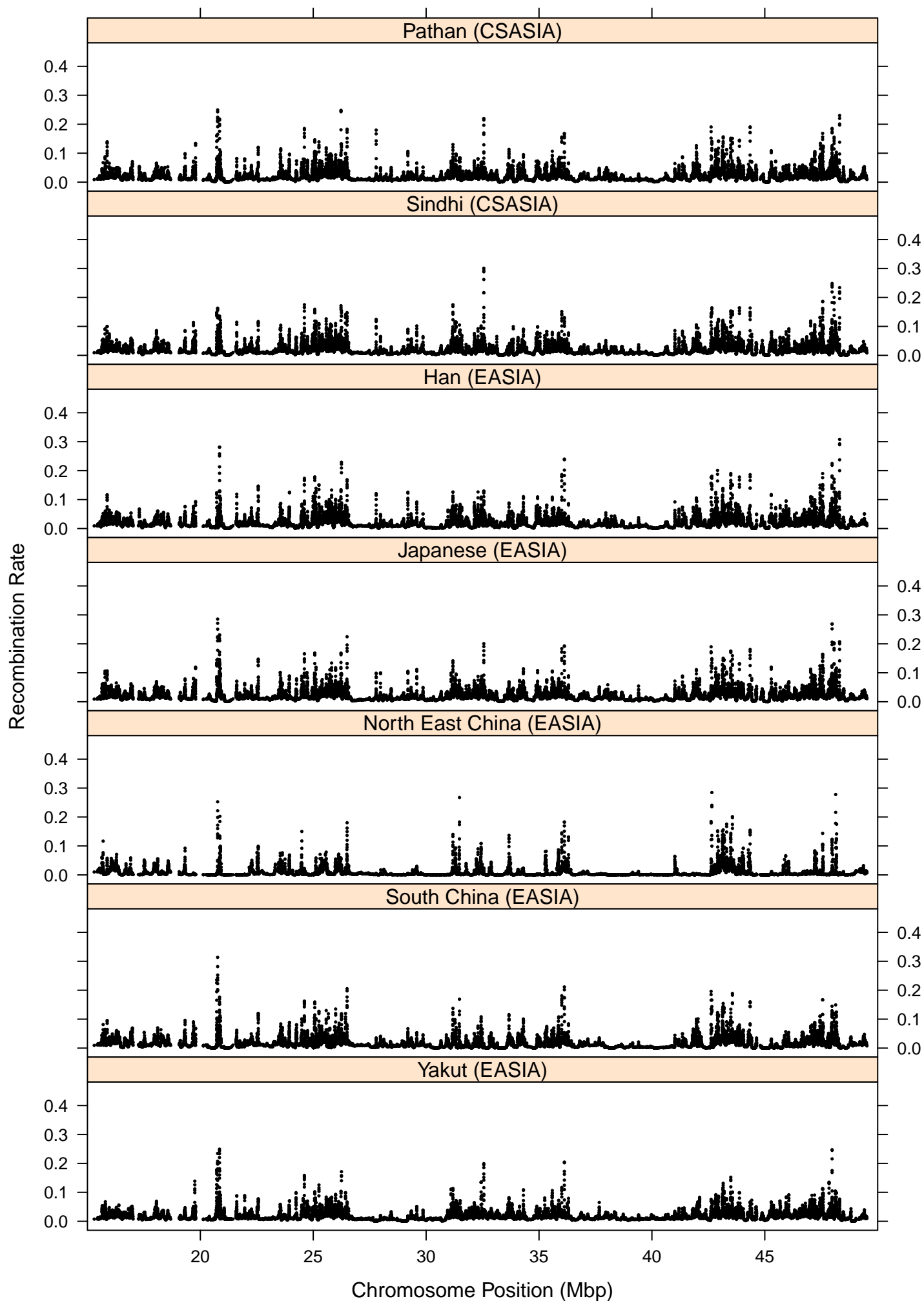

Supplement: Figure S1 — Recombination rate estimates (4Ner/kb) corrected for effective population size for successive SNP-pairs for chromosome 22 and in each of 28 populations, grouped into geographical regions. (PDF) [file pone.0017913.s001.pdf]

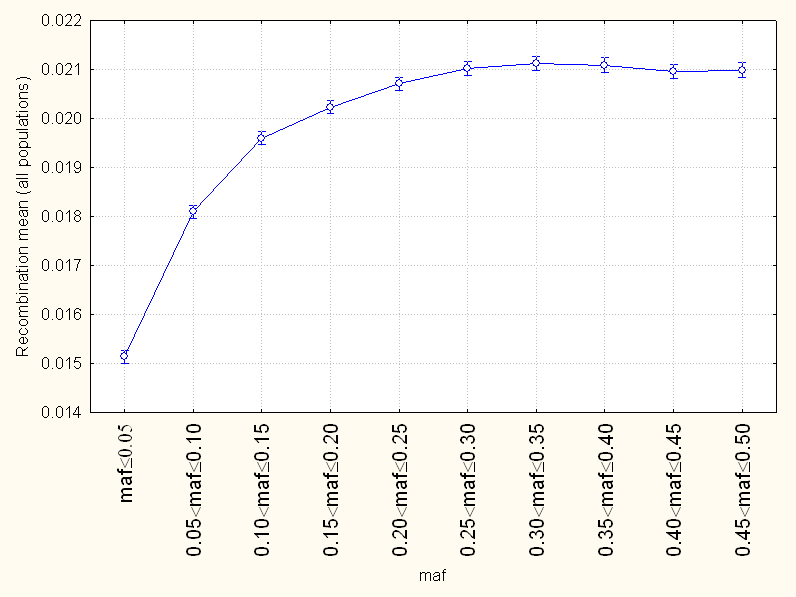

Supplement: Figure S2 — Mean of the recombination estimate (4Ner/kb) for all populations calculated for 10 categories of SNPs based on their minor allele frequency. MAFs are calculated using the global allele frequency of all populations together. (TIF) [file pone.0017913.s002.tif]

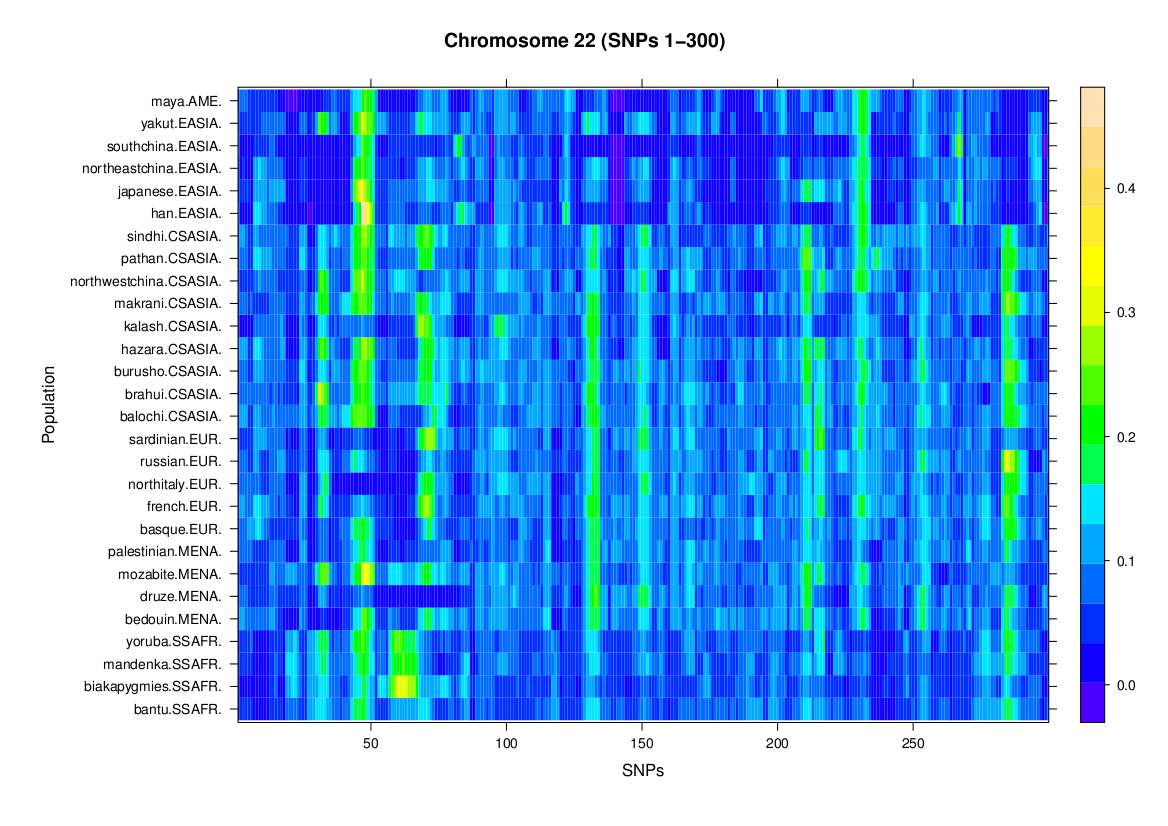

Supplement: Figure S3 — Heatmap showing patterns of hotspots observed for 300 SNPs of chromosome 22 for the 28 populations, grouped according to their geographical region. The first 300 SNPs of chromosome 22, for which a hotspot is present in at least one population, are reported on the x axis. In color, for each population the value of the recombination estimate (4Ner/kb) corrected for effective population size for that SNP in a gradient from blue (low recombination values) to green (high recombination values). (TIF) [file pone.0017913.s003.tif]
