# Supplementary material for: Similarity in Recombination Rate Estimates Highly Correlates with Genetic Differentiation in Humans
Source: PLoS One. 2011 Mar 28;6(3):e17913. doi: 10.1371/journal.pone.0017913 (PMC3065460; doi:10.1371/journal.pone.0017913)
Supplement: Table S1 — Mantel's r values between the genetic distance and recombination dissimilarity matrices. First row shows chromosome for which the genetic distance was calculated; first column show the chromosome for which the recombination matrix was calculated. All value were highly significant (p<0.00001). (DOC) [file pone.0017913.s004.doc]

***Table S1***

|  | 1 | 2 | 3 | 4 | 5 | 6 | 7 | 8 | 9 | 10 | 11 | 12 | 13 | 14 | 15 | 16 | 17 | 18 | 19 | 20 | 21 | 22 | mean |
| --- | --- | --- | --- | --- | --- | --- | --- | --- | --- | --- | --- | --- | --- | --- | --- | --- | --- | --- | --- | --- | --- | --- | --- |
| 1 | 0.91 | 0.92 | 0.91 | 0.90 | 0.92 | 0.91 | 0.91 | 0.90 | 0.91 | 0.92 | 0.91 | 0.92 | 0.92 | 0.91 | 0.91 | 0.90 | 0.89 | 0.89 | 0.90 | 0.89 | 0.90 | 0.92 | 0.91 |
| 2 | 0.90 | 0.91 | 0.90 | 0.89 | 0.90 | 0.90 | 0.90 | 0.88 | 0.90 | 0.91 | 0.90 | 0.90 | 0.90 | 0.89 | 0.90 | 0.88 | 0.87 | 0.88 | 0.89 | 0.87 | 0.89 | 0.91 | 0.89 |
| 3 | 0.85 | 0.86 | 0.85 | 0.84 | 0.86 | 0.85 | 0.85 | 0.84 | 0.86 | 0.86 | 0.85 | 0.86 | 0.86 | 0.85 | 0.85 | 0.83 | 0.82 | 0.83 | 0.84 | 0.83 | 0.83 | 0.86 | 0.85 |
| 4 | 0.90 | 0.91 | 0.90 | 0.90 | 0.91 | 0.91 | 0.91 | 0.89 | 0.91 | 0.91 | 0.91 | 0.91 | 0.91 | 0.90 | 0.90 | 0.89 | 0.88 | 0.89 | 0.90 | 0.88 | 0.90 | 0.92 | 0.90 |
| 5 | 0.90 | 0.91 | 0.90 | 0.89 | 0.91 | 0.91 | 0.90 | 0.89 | 0.90 | 0.91 | 0.91 | 0.91 | 0.91 | 0.90 | 0.90 | 0.89 | 0.88 | 0.88 | 0.89 | 0.88 | 0.89 | 0.91 | 0.90 |
| 6 | 0.92 | 0.93 | 0.93 | 0.92 | 0.93 | 0.93 | 0.93 | 0.92 | 0.93 | 0.93 | 0.93 | 0.93 | 0.93 | 0.93 | 0.92 | 0.91 | 0.91 | 0.91 | 0.92 | 0.91 | 0.92 | 0.94 | 0.92 |
| 7 | 0.89 | 0.90 | 0.89 | 0.88 | 0.90 | 0.89 | 0.89 | 0.88 | 0.89 | 0.90 | 0.89 | 0.89 | 0.90 | 0.89 | 0.89 | 0.87 | 0.86 | 0.87 | 0.88 | 0.87 | 0.88 | 0.90 | 0.89 |
| 8 | 0.86 | 0.88 | 0.86 | 0.85 | 0.87 | 0.87 | 0.87 | 0.85 | 0.87 | 0.88 | 0.87 | 0.87 | 0.87 | 0.86 | 0.86 | 0.85 | 0.84 | 0.84 | 0.85 | 0.84 | 0.85 | 0.88 | 0.86 |
| 9 | 0.89 | 0.90 | 0.89 | 0.88 | 0.90 | 0.89 | 0.89 | 0.88 | 0.89 | 0.90 | 0.89 | 0.89 | 0.90 | 0.88 | 0.88 | 0.87 | 0.87 | 0.87 | 0.88 | 0.87 | 0.87 | 0.90 | 0.88 |
| 10 | 0.94 | 0.94 | 0.94 | 0.93 | 0.94 | 0.94 | 0.94 | 0.93 | 0.94 | 0.95 | 0.94 | 0.94 | 0.94 | 0.93 | 0.93 | 0.93 | 0.92 | 0.92 | 0.93 | 0.92 | 0.93 | 0.95 | 0.93 |
| 11 | 0.91 | 0.92 | 0.92 | 0.91 | 0.92 | 0.92 | 0.92 | 0.90 | 0.92 | 0.93 | 0.92 | 0.92 | 0.92 | 0.91 | 0.91 | 0.90 | 0.89 | 0.90 | 0.91 | 0.89 | 0.91 | 0.93 | 0.91 |
| 12 | 0.88 | 0.89 | 0.88 | 0.87 | 0.89 | 0.89 | 0.88 | 0.87 | 0.88 | 0.90 | 0.89 | 0.89 | 0.89 | 0.88 | 0.88 | 0.87 | 0.85 | 0.86 | 0.88 | 0.86 | 0.88 | 0.90 | 0.88 |
| 13 | 0.87 | 0.88 | 0.87 | 0.86 | 0.87 | 0.87 | 0.87 | 0.85 | 0.87 | 0.88 | 0.87 | 0.87 | 0.88 | 0.87 | 0.86 | 0.85 | 0.84 | 0.85 | 0.86 | 0.85 | 0.85 | 0.88 | 0.86 |
| 14 | 0.85 | 0.86 | 0.85 | 0.84 | 0.86 | 0.85 | 0.85 | 0.84 | 0.86 | 0.86 | 0.85 | 0.86 | 0.86 | 0.85 | 0.85 | 0.83 | 0.82 | 0.83 | 0.84 | 0.83 | 0.83 | 0.86 | 0.85 |
| 15 | 0.88 | 0.90 | 0.89 | 0.87 | 0.89 | 0.89 | 0.89 | 0.87 | 0.89 | 0.89 | 0.89 | 0.89 | 0.89 | 0.88 | 0.88 | 0.87 | 0.86 | 0.87 | 0.87 | 0.86 | 0.87 | 0.89 | 0.88 |
| 16 | 0.78 | 0.79 | 0.78 | 0.76 | 0.78 | 0.78 | 0.78 | 0.76 | 0.79 | 0.79 | 0.77 | 0.78 | 0.79 | 0.77 | 0.77 | 0.76 | 0.75 | 0.75 | 0.76 | 0.76 | 0.75 | 0.79 | 0.77 |
| 17 | 0.90 | 0.91 | 0.90 | 0.89 | 0.91 | 0.90 | 0.90 | 0.89 | 0.91 | 0.91 | 0.90 | 0.91 | 0.91 | 0.90 | 0.89 | 0.89 | 0.89 | 0.89 | 0.90 | 0.89 | 0.89 | 0.91 | 0.90 |
| 18 | 0.90 | 0.91 | 0.89 | 0.88 | 0.90 | 0.89 | 0.90 | 0.88 | 0.90 | 0.90 | 0.89 | 0.90 | 0.90 | 0.89 | 0.89 | 0.88 | 0.87 | 0.88 | 0.88 | 0.88 | 0.88 | 0.90 | 0.89 |
| 19 | 0.82 | 0.83 | 0.82 | 0.80 | 0.82 | 0.82 | 0.82 | 0.80 | 0.82 | 0.83 | 0.82 | 0.82 | 0.83 | 0.81 | 0.81 | 0.80 | 0.79 | 0.80 | 0.80 | 0.79 | 0.80 | 0.83 | 0.81 |
| 20 | 0.90 | 0.90 | 0.89 | 0.88 | 0.90 | 0.89 | 0.89 | 0.88 | 0.90 | 0.90 | 0.89 | 0.90 | 0.90 | 0.89 | 0.89 | 0.88 | 0.87 | 0.87 | 0.88 | 0.87 | 0.87 | 0.90 | 0.89 |
| 21 | 0.88 | 0.89 | 0.88 | 0.87 | 0.89 | 0.89 | 0.89 | 0.87 | 0.88 | 0.89 | 0.89 | 0.89 | 0.89 | 0.88 | 0.89 | 0.87 | 0.86 | 0.87 | 0.88 | 0.86 | 0.88 | 0.90 | 0.88 |
| 22 | 0.80 | 0.81 | 0.80 | 0.79 | 0.81 | 0.81 | 0.80 | 0.78 | 0.80 | 0.81 | 0.80 | 0.81 | 0.81 | 0.80 | 0.79 | 0.78 | 0.77 | 0.77 | 0.79 | 0.77 | 0.79 | 0.82 | 0.80 |
| mean | 0.88 | 0.89 | 0.88 | 0.87 | 0.89 | 0.88 | 0.88 | 0.87 | 0.88 | 0.89 | 0.88 | 0.89 | 0.89 | 0.88 | 0.88 | 0.86 | 0.85 | 0.86 | 0.87 | 0.86 | 0.87 | 0.89 |  |
